# Understanding the Knowledge, Attitudes, and Practices Concerning Sugar-Sweetened Beverages and Beverage Taxation among Saudi University Students

**DOI:** 10.3390/nu15194151

**Published:** 2023-09-26

**Authors:** Noor M. Alothmani, Noha M. Almoraie

**Affiliations:** Department of Food and Nutrition, Faculty of Human Sciences and Design, King Abdulaziz University, Jeddah 3270, Saudi Arabia; nabdullahalothmani@stu.kau.edu.sa

**Keywords:** sugar-sweetened beverages, knowledge, attitudes, practices, tax, university students

## Abstract

University students should be informed about sugar-sweetened beverages (SSBs). Given the high consumption level of SSBs in Saudi Arabia, this study aims to examine the knowledge, attitudes, and practices related to SSBs and taxes. This cross-sectional study was conducted on 380 students at King Abdulaziz University in Jeddah between September 2022 and March 2023. The data were collected using an online self-administered questionnaire. The results reveal that 93% of the students had high knowledge scores, 99% have positive attitudes, and 37% present good practices toward SSBs. Moreover, 73% of students have high knowledge scores, 83% have positive attitudes, and 32% present good practices regarding SSB taxes. Each of these variables, college education, smoking, family income, body mass index, activities, and sports, showed statistically significant differences between gender with regard to the scores for knowledge, attitudes, and practice towards SSBs and their taxes. Thus, a high percentage of university students present sufficient knowledge of and good attitudes towards SSBs and taxation, unlike the practical results. Policymakers should develop strategic approaches and education campaigns to improve practices towards SSBs.

## 1. Introduction

Sugar-sweetened beverages (SSBs) have been identified as major contributors to the global burden of non-communicable diseases, including obesity, diabetes, and cardiovascular diseases. In Saudi Arabia, the prevalence of these diseases is high and SSB consumption is a significant public health concern. According to the World Health Organization (WHO), in 2016, more than 1.9 billion people aged 18 years and older were overweight, including 650 million obese adults [1]. Notably, the rates of being overweight and obese are increasing significantly in Saudi Arabia, one of the most obese countries worldwide [2]. According to the National Nutrition Survey in 2021, the overall obesity prevalence rate will reach 33.7% in Saudi Arabia, which ranks fifteenth among the countries with the highest prevalence of obesity [3]. Based on an estimate of the consumption of SSBs from the recent research, SSBs are very common in Saudi Arabia. Specifically, 75% of Saudis regularly consume SSBs, which are a major dietary source of added sugars in the country [4]. Moreover, in a study of 1194 adults in Saudi Arabia, it was reported that 85% of adults consumed SSBs [5]. Likewise, studies conducted in Dammam, Imam Abdulrahman University, which included 507 students, demonstrated that the average volume of consumed SSBs was >4 L/week, equivalent to 650 mL/day [6]. Additionally, a recent study was conducted in Saudi Arabia, where 10,118 participants were recruited from the 2013 Saudi Health Interview Survey, reporting that about 71% of them, or three out of four individuals, consumed SSBs at least once a week [7]. Several studies have shown that SSBs are a controversial dietary component in public health discussions worldwide. Consuming large amounts of SSBs is associated with excess sugar levels and calories, and thus, weight gain and obesity [8,9,10]. Furthermore, one of the most important aspects of making health decisions is having an adequate knowledge of the contents of foods and beverages and their associated health-related consequences [11]. Specifically, the literature indicates that an adequate knowledge of sugar content and calories in SSBs, as well as health risks, is associated with changing attitudes towards SSBs and lowering SSB consumption levels [12,13,14]. The more nutritional knowledge individuals have about SSBs, the lower their consumption. The knowledge and attitudes towards SSBs are often measured according to three categories of information: the amount of sugar and calories in SSBs, the daily permissible consumption level of SSBs, and the effect of an excessive consumption of SSBs on health [11]. Measuring individuals’ knowledge about SSBs is critical for assessing their choice to consume SSBs and the consumption rate [15]. Dietary patterns and individuals’ attitudes towards SSB consumption are influenced by many factors, such as knowledge, geographic area, and income level [16]. Specifically, several studies have reported that SSB consumption is higher among individuals with a low socioeconomic status [16,17]. This is often due to their limited knowledge of the sugar and calorie contents of these SSBs, as well as the fact that foods with added sugar and high calories are less expensive than healthy foods [17]. A study conducted in Los Angeles assessed the knowledge, nutritional attitudes, and their relationship with SSB consumption through surveys. The results of this study indicated that individuals with sufficient knowledge of the calorie content of SSBs consumed fewer SSBs than others [18]. In contrast, one such study was conducted among university students in Nigeria. The results indicated that the students had an adequate perception and knowledge of SSBs and their health consequences; however, the consumption of SSBs was high among university students. This may be due to their perception that SSBs are social drinks and because they are abundantly available at the university [19]. In Saudi Arabia, attitudes towards SSB consumption levels were assessed in a cross-sectional study conducted among Saudi women. Many Saudi women admitted that SSBs were inappropriate and harmful to their health; however, they were considered necessary while eating and in social gatherings, leading to high consumption rates [20]. In another study conducted in Medina, Saudi Arabia, to assess adults’ knowledge, attitudes, and behaviors towards SSBs, most participants were aware of the harm to their health from excessive SSB consumption. Moreover, the marked increase in knowledge may be due to health education programs focusing on improving living habits and the quality of life [8]. An adequate knowledge of the content and health consequences of SSBs is often considered a prerequisite for behavioral modification and healthy decision making. Attitudes towards SSBs have also been described as important consumption indicators [11].

There is a need to increase health awareness and education regarding SSBs and their health consequences to reduce SSB consumption among community members. Taxes have been widely implemented to reduce the consumption of SSBs and encourage the selection of healthy alternatives [8]. With this intended aim, the WHO recommended a 20% tax rate on SSBs. Many countries, including the United States, France, England, and Mexico, have implemented these taxes [10]. In 2017, the General Authority of Zakat and Tax in Saudi Arabia imposed a 100% tax on energy drinks and 50% on soft drinks [21]. In the United States, the expected response to the SSB tax was assessed as 20% through a survey, with the results indicating that 36% of the respondents supported the introduction of an SSB tax, with greater support from those aged 18–24 years old, those with a BMI of 30 kg/m^2^, and those with higher educational levels [22]. Similarly, a systematic review concluded that an additional tax on SSBs was an effective policy that may reduce SSB consumption among social groups. Furthermore, this review demonstrates that taxes on SSBs have a greater positive impact on those with lower incomes in the community [23]. Young adults in universities represent a large proportion of the community and are an interesting group to study alongside universities that support health promotion initiatives. Measuring their knowledge and attitudes towards SSBs is important because they represent a significant group that needs to be educated and gain an increased awareness of SSBs and their health consequences. In addition, it is important to assess the public’s support for the SSB tax and examine university students’ reactions to it. This study aims to examine the knowledge, attitudes, and practices (KAPs) toward SSBs and SSB tax among healthy young adults at King Abdulaziz University, Jeddah, Saudi Arabia.

## 2. Materials and Methods

### 2.1. Study Design and Participants

We used a cross-sectional design to achieve our study objectives. This quantitative and descriptive study evaluated the knowledge and attitudes towards SSBs and SSB taxation among healthy young adults at King Abdulaziz University, Jeddah, Saudi Arabia, between September 2022 and March 2023. This study was approved by the Committee on the Ethics of Human Research of the Faculty of Medicine at King Abdulaziz University, Jeddah, Saudi Arabia (Reference No. 251-22). All participants signed an informed consent form. The informed consent form contained information about the study’s aims, purposes, and procedures. This indicated that the participants would not be harmed and that the information would be treated as strictly confidential and used only for scientific research. The participants were encouraged to ask questions if they did not understand anything or needed clarification of the information on the informed consent form. Participants were also informed of their rights to accept or refuse participation and their ability to withdraw at any time while answering the questionnaire. According to the Deanship of Admission and Registration at King Abdulaziz University, the total student population was approximately 80,000 in 2019 [24]. Thus, the sample size calculator indicated that the sample size needed to achieve sufficient statistical power was 270, which was based on a 5% margin of error, 90% confidence level, and response distribution of 50% [25]. The participants were recruited from King Abdulaziz University in Jeddah, Saudi Arabia. The study sample comprised healthy, young, Saudi participants of both genders (aged 18–25 years). The exclusion criteria were the presence of any disease (such as diabetes, cardiovascular disease, hypertension, cancer, or eating disorders) and pregnancy or lactation. This study used a random sample of university students from all academic years.

### 2.2. Data Collection

The invitation to participate in the survey was sent via e-mail to students and through social networking sites. The e-mail included an introduction to the questionnaire explaining the aim of the study and guaranteeing anonymity. The researcher collected study data and anthropometric measurements. The questionnaire, consisting of four sections is attached in the Supplementary Materials. Specifically, the first section comprised 13 questions aimed at assessing the university students’ social and demographic characteristics, while the second section aimed to determine the KAPs towards SSBs among the participating students at King Abdulaziz University. The questionnaire in this section discussed three themes, namely, the domain of KAPs, consisting of 31 questions about the participants’ KAPs regarding sugar-sweetened beverages, which were adopted from Rivard et al. [22] and Teng et al. [26]. The third section examined the participants’ KAPs towards the SSB tax. Furthermore, the assessment of KAPs towards taxing SSBs, which was conducted using 10 questions, was adapted and modified from 2 prior studies: Rivard et al. [22] and Baijnath [27]. These adaptations were specifically performed to align with the objectives and population of the present study. Additionally, anthropometric measurements were performed using the Quetelet equation (body mass index (BMI): weight (kg)/height (m^2^)). Self-reported height and weight data were used to measure the BMI and described according to the WHO criteria. Four categories were identified: underweight (BMI < 18.5 kg/m^2^), normal weight (18.5 kg/m^2^ ≤ BMI < 25.0 kg/m^2^), overweight (25.0 kg/m^2^ ≤ BMI < 30.0 kg/m^2^), and obesity (BMI ≥ 30.0 kg/m^2^) [28]. A pilot questionnaire was used to identify content, language, or layout issues. A small sample of ten people, excluded from the study population, was given a copy of the first version of the questionnaire and invited to provide feedback regarding its ease of use to obtain information concerning any issues or difficulties that subsequent respondents may encounter. The feedback was integrated into the final version of the questionnaire. The data from the pilot study were excluded from the analysis. However, a pilot study was conducted to ascertain the sample size, method of data collection, validity, and clarity of the questionnaire, and to estimate the time required to complete the questionnaire.

### 2.3. Knowledge, Attitudes, and Practice Scores Concerning Sugar-Sweetened Beverages

The KAP section towards SSBs consisted of 31 dichotomous questions (18 items on knowledge, 6 items on attitudes, and 7 items on practice) that required answering yes/no or agree/disagree. To be considered eligible for KAPs concerning SSBs, the participants had to score higher than 50% of the total score, as the total score was calculated by summing the correct answers from the participants. The maximum score for each domain of the KAP was equal to the number of items; therefore, a higher score reflected a higher indicator and vice versa. Moreover, each domain was classified into two levels of indicators, “high” or “low”, where an oasis point was awarded for the correct answer and zero points for the incorrect answer. In the domain of knowledge, “low knowledge” was indicated if the score was (0–8), while “high knowledge” was indicated if the score was (9–18). Similarly, a “negative attitude” was indicated if the score was (0–2) and a “positive attitude” was indicated if the score was (3–6). Moreover, in the practice domain, “poor practice” was indicated if the scores were (0–3), while “good practice” was indicated if the scores were (4–7) [15,26].

### 2.4. Knowledge, Attitudes, and Practice Scores for the Tax on Sugar-Sweetened Beverages

The questions related to knowledge, attitudes, and practices concerning the tax on SSBs consisted of 10 items (4 items on knowledge, 4 on attitudes, and 2 on practice). Questions 4 to 7 in the knowledge section contained five possible answers where the responses were categorized into three levels (“strongly agree and agree”, “neutral”, and “strongly disagree or disagree”). Two points were awarded for “strongly agree and agree”, one point for “neutral”, and zero points for “strongly disagree and disagree”. Similarly, in the attitudes section, the first question had three possible answers: “Replace it with non-taxable drinks (such as fresh fruit juices, water, or unflavored milk)”, “Reduce consumption of SSBs (such as soft drinks and energy drinks)”, and “There is no change at all”. One point was awarded for the correct answer, “Replace it with non-taxable drinks (such as fresh fruit juices, water, or unflavored milk),” and zero points were given for the remaining answers. The second and fourth questions from the attitudes section had five possible answers, and the responses were categorized into three levels (“strongly agree and agree”, “neutral”, and “strongly disagree or disagree”). Two points were awarded for “strongly agree and agree”, one point for “neutral”, and zero points for “strongly disagree and disagree”. The third question included four possible answers: “Fundamental obesity interventions”, “Revenue for the government”, “Subsidizing healthy foods”, and “Researching cures for diseases exacerbated by excess sugar consumption”. One point was awarded for a possible correct answer. The ninth question of the practice section included four possible answers: “Continue to buy SSBs in the same quantity and with the same frequency that I did before the tax was introduced”, “Still buy SSBs, but less often”, “Stopped buying SSBs and would rather buy some other beverage”, and “This does not apply to me, as I do not buy SSBs at all”. One point was awarded for the correct answer, “Stopped buying SSBs and would rather buy some other beverage”, and zero points for the remaining answers. Finally, the tenth question had six possible answers: “This does not apply to me, as I do not buy SSBs anyway”, “Milk and milk products”, “100% fruit juice”, “Sugar-free drink options”, “Water”, and “Other”. One point was awarded for a possible correct answer. The degree of knowledge ranged from (0–8); therefore, knowledge was classified as low if it was (0–3) and high if it was (4–8). Similarly, the degree of attitudes was 0–6, where attitudes were classified as negative if they were (0–2) and positive if (3–6). The degrees of practice were (0–2), as the practice was classified as poor practice if it was (0–1) and good practice if it was (2) [22,27].

### 2.5. Statistical Analyses

All statistical analyses were performed using SPSS statistical analysis software (version 23). The socio-demographic characteristics, health data, and answers pertaining to the knowledge of and attitudes and practices towards SSBs and SSB taxation were presented as frequencies and percentages. University students’ KAPs regarding SSBs and the taxation questions and responses are presented in Tables S1–S6. Additionally, the scores pertaining to the knowledge of and attitudes towards SSBs and SSB taxation were presented as mean ± SD. Chi-squared tests were used to examine the associations between the socio-demographic characteristics and knowledge of and attitudes towards SSBs and SSB taxation. Furthermore, a linear regression was conducted to examine the association between the socio-demographic characteristics, knowledge of, and attitudes towards SSBs and SSB taxation scores. Differences were considered statistically significant at *p* < 0.05.

## 3. Results

### 3.1. Socio-Demographic Characteristics and Health Data

The socio-demographic characteristics and health data of university students stratified by sex are presented in Table 1. This study included 380 university students, of whom 184 and 196 were males and females, respectively. Most of the students (36%) were aged 18–19 years (34% male and 37% female) and (35%) of the students were aged 20–21 years (41% males and 30% females). The vast majority of students were single (96%) (97% male and 94% female), in their first academic year (foundation year) (39%), studying in the science department (33%), humanities and language department (27%), and foundation year (27%). In addition, 95% of the students lived with their families and the average family income was SAR >5000 per month (28%). Importantly, income in Saudi Arabia is categorized in terms of Saudi riyals (SAR), being classified as low and high if it is less than SAR 5000 and exceeds SAR 20,000, respectively [29]. Most students (83%) reported that they did not smoke, while 36% exercised 1–2 times a week. Additionally, 34% of the students stated that they did not exercise at all. The results show that 85% of the students sleep at night and 71% sleep for 5–8 h per day. Furthermore, 56% of the students ate 2 meals per day. Regarding the BMI, the majority of students (54%) had a normal weight. There were statistically significant differences between sexes in college, smoking, exercise, meal intake, and BMI. The number of male students in the environmental sciences faculty was higher than that of female students, while the number of female students was higher in the health sciences, humanities, and language faculties, and foundation year (*p* = 0.000). The percentage of male students who smoked was higher than that of female students (*p* = 0.000). The percentage of male students who exercised > 4 times a week was higher than that of female students, while the percentage of female students who did not exercise at all (none) was higher than that of male students (*p* = 0.007). Regarding eating meals, the percentage of male students was higher when eating three meals per day; in contrast, the percentage of female students was higher when eating one or two meals per day (*p* = 0.001). In terms of body mass index, the percentage of male students was higher in the overweight and obese categories, while the percentage of female students was higher in the underweight and normal weight categories (*p* = 0.004).

### 3.2. Factors Associated with a Knowledge of and Attitudes and Practices towards Sugar-Sweetened Beverages

Table 2 presents the socio-demographic characteristics associated with university students’ KAPs concerning SSBs. Female students were found to be more knowledgeable about SSBs than male students; however, the difference was not statistically significant. It was also found that students in the science faculty had the highest positive attitudes towards SSBs (33%), followed by students in the humanities and language faculty and the foundation year, followed by the health sciences and environmental sciences faculties (28%, 28%, 23%, and 20%, respectively) (*p* = 0.026). In addition, students who did not smoke had more positive attitudes towards SSBs (84%) than those who smoked (16%) (*p* = 0.009). Moreover, students with a family income SAR < 5000 per month had better practices towards SSBs (34%) than students with a family income of SAR 5000 to less than 10,000 (24%), 10,000 to less than 15,000 (20%), >20,000 (13%), and 15,000–20,000 (9%) (*p* = 0.009). Generally, about (93%) of the students had high knowledge levels concerning SSBs, (99%) of the students had positive attitudes towards SSBs, and (37%) of the students had good practices towards SSBs.

### 3.3. Factors Associated with the Knowledge of and Attitudes and Practices towards Sugar-Sweetened Beverages Taxation

Table 3 presents the socio-demographic characteristics associated with university students’ knowledge of and attitudes towards SSB taxation. Female students were more knowledgeable about SSB taxation (56%) than the male students (44%) (*p* = 0.007). Similarly, female students had more positive attitudes toward SSB taxation (52%) than the male students (48%); however, this difference was not statistically significant. Furthermore, non-smoking students had better practices toward SSB taxation (93%) than smokers (7%) (*p* = 0.000). In addition, students who exercised 1–2 times per week (41%) had better practices toward SSB taxation compared to those who did not exercise (none), exercised 3–4 times per week, and exercised >4 times per week (23%, 22%, and 14%, respectively) (*p* = 0.015). Overall, about (73%) of the students had high knowledge scores for SSB taxation, (82%) of the students had positive attitudes towards SSB taxation, and 32% of the students had good practices towards SSB taxation.

Table 4 presents the association between the knowledge of and attitudes towards SSBs and the selected socio-demographic factors for university students stratified by sex. The knowledge scores show a positive relationship with activities or sports among female students (*p* = 0.010). The attitude scores positively correlate with colleges among male students (*p* = 0.004). The attitude scores show a positive relationship with smoking among male students (*p* = 0.022). Moreover, the practice scores are positively related to family income among female students (*p* = 0.017).

Table 5 presents the association between the scores for KAPs concerning SSB taxation and the selected socio-demographic factors for university students stratified by gender. The knowledge scores are positively related to BMI among male students (*p* = 0.029). There are no statistically significant relationships with attitudes. Furthermore, practice scores indicate a positive relationship with smoking for both males and females (*p* = 0.016 and 0.010, respectively). The practice scores indicate a positive relationship with activities or sports among male students (*p* = 0.050).

Linear regression was used to examine the association between the socio-demographic characteristics, BMI, and knowledge, attitude, and practices concerning sugar-sweetened beverage taxation scores; a *p*-value of 0.05 was considered statistically significant. 

## 4. Discussion

Several studies have shown that SSBs are a controversial dietary component in public health discussions worldwide. The consumption of high levels of SSBs is associated with excess sugar and calories levels, weight gain, obesity, and many non-communicable diseases [8,9,10]. The rates of being overweight and obese have also increased in Saudi Arabia, one of the world’s fattest countries, ranking 15th worldwide [2]. Young university students represent a large proportion of society and are an interesting group to study alongside universities that support health promotion initiatives. Thus, university students must have high levels of knowledge of, positive attitudes, and good practices towards SSBs and taxes. This study aimed to examine the KAPs concerning SSBs and SSB taxes among healthy young adults at King Abdulaziz University, Jeddah, Saudi Arabia. The study revealed that the number of male university students in the faculties of science and environmental sciences was higher than that of female students, while the number of female students was higher in the faculties of health sciences, humanities, and languages, and in the foundation year. The number of male students who smoked was higher than that of female students. The percentage of male students who exercised > 4 times a week was higher than that of female students, whereas the percentage of female students who did not exercise at all was higher than that of male students. As for eating meals, the percentage of male students who ate three meals a day was higher than that of female students, whereas the percentage of female students who ate one or two meals a day was higher. For BMI, the number of male students was higher in the overweight and obese categories, whereas the percentage of female students was higher in the underweight and normal weight categories. Overall, this study revealed high levels of knowledge, positive attitudes, and poor practices concerning SSBs. Regarding the socio-demographic characteristics associated with students’ KAPs concerning SSBs, science college students had the most positive attitudes toward SSBs compared to students at other colleges. In addition, students who did not smoke had more positive attitudes toward SSBs than those who smoked. Moreover, students with a family income lower than SAR 5000 per month had better practices towards SSB consumption than those in other income groups. The study revealed a moderate knowledge of, positive attitudes, and poor practices toward the SSB tax. Female students were more aware of SSB taxation than were male students. Non-smoking students also had better practices toward the SSB tax than smoking students. In addition, students who exercised once or twice a week had higher rates of good practices than other students. Hence, the results indicate that the degree of knowledge related to SSBs positively correlates with activities or sports among female students. The attitude scores showed a positive relationship between college education and smoking among male students. Additionally, there was a positive relationship between practice scores and family income among female students. Regarding the knowledge scores related to SSB tax, the results show a positive relationship with BMI among male students. The practice scores also indicated a positive relationship with activities or sports among male students only.

One of the most important aspects of making healthy decisions is having an adequate knowledge of the contents of foods and beverages and their health consequences [18]. The evidence shows that nutrition knowledge plays a major role in the adoption of healthy eating habits [30]. An essential part of improving one’s health is learning to consume less fat, salt, and sugar commonly found in processed foods and SSBs [31,32]. High knowledge scores for, positive attitudes, and good practices toward SSBs among university students are critical. In the current study, a high level of knowledge was observed among university students; specifically, approximately 93% of the students had high knowledge scores for SSBs. The measure of knowledge related to SSBs was represented in statements that revolved around the forms and content of sugars in SSBs and the health consequences that resulted from the frequent consumption of SSBs. Our results show a high level of knowledge, which may be attributed to the efforts exerted in providing activities and educational programs that focus on healthy lifestyles, in addition to the efforts of universities to provide adequate information through educational content for students. According to Mazier and Macleod, a single nutrition course can improve undergraduate students’ nutritional knowledge [33]. Therefore, nutrition education is likely to increase students’ knowledge and improve their healthy eating habits. The results of our study, in terms of a greater knowledge of SSBs among university students, are consistent with those of several other studies. A study conducted in Los Angeles showed a high level of knowledge about SSBs and a low consumption rate of SSBs [18]. In contrast, some studies have shown good levels of knowledge among university students; however, despite this, the consumption of SSBs was high among students [19,34,35]. This may be because these beverages are widely available in universities and are perceived as social drinks; thus, peer influence plays an important role. Similarly, the study indicated positive attitudes towards SSBs among university students, with 99% reporting positive attitudes regarding SSBs. Attitudes towards SSBs among university students were measured based on their agreement or disagreement with the statements related to SSBs, where the students showed a very positive attitude. The level of attitude in the current study was similar to a study conducted in the United Arab Emirates, where the results indicated that 77% of university students showed positive attitudes toward added sugar; however, despite this, some of them mentioned that their appetite or taste may have affected their consumption levels [36]. This result was consistent with the previous work conducted in Saudi Arabia to assess Saudi women’s attitudes towards SSBs, in which SSBs were considered inappropriate and harmful to health but were reported to be consumed when necessary, during eating and social gatherings [20]. This may be attributed to the fact that social gatherings are common among Saudi individuals and are usually characterized by consuming unhealthy foods and drinks.

Regarding practices, the results show poor SSB practices among university students, with only 37% of students following good practices towards SSBs. Practices related to SSBs were measured through phrases, such as determining the amount of sugar added to beverages before consuming them, preference for SSBs over unsweetened beverages, consumption of SSBs when watching TV and in meetings with family and friends, and others. Although the levels of knowledge of and attitudes towards SSBs were high in the current study, the practice score for SSBs was poor and unacceptable. This result is consistent with a similar study conducted in Malaysia, where the results show good attitudes, moderate knowledge of, and poor practices related to SSBs [15]. Moreover, this is consistent with a previous work conducted in Lahore among female students at the College of Health, where the results showed sufficient knowledge of soft drinks and their effects on health, although the participants’ consumption was high and their practices did not promote health [34]. Interestingly, university students’ nutritional knowledge does not necessarily lead to healthy eating habits. Stockton and Baker found that college students were aware of the harm caused by eating fast food; however, despite this, their knowledge did not affect their food choices [37], potentially due to their knowledge of and attitudes towards these foods preventing them from changing their behavior. According to a qualitative study, various personal, cultural, environmental, and academic factors affect college students’ dietary habits. Peer influence, accessibility, and food costs are social and environmental factors [38]. The ease of access to SSBs on campus and their consumption with peers, in addition to the fact that SSBs have a pleasant taste that stimulates consumption habits, especially in the summer when they are cold and refreshing, may explain the poor practices related to SSBs in the current study, despite the level of sufficient knowledge and positive attitudes among students. The present study indicated that 73% of university students had high knowledge scores, 82% had positive attitudes, and 32% had good practices towards SSB taxation. The knowledge, attitudes, and practices related to SSB taxation were measured through phrases, such as the purpose of the tax, where its revenues go, attitudes towards this tax, related practices, and the alternatives consumed instead of beverages after applying the tax. The evidence from a South African study showed that the majority of participants (58%) had a sufficient knowledge of the SSB tax and their attitudes toward the SSB tax varied. Only 8.1% of the participants indicated that they would stop buying SSBs and replace them with other drinks. About one-third of the participants stated that they would not stop consuming SSBs and that they may consume them in reduced quantities [27]. This result is somewhat consistent with the students’ practice scores concerning the tax on SSBs in the current study. Furthermore, the current evidence is somewhat consistent with two previous studies conducted in Medina, where the first study indicates that 56% of the participants support a tax on energy drinks and 60% support a tax on soft drinks. The participants also reduced their consumption of these drinks and replaced them with water [8]. Moreover, the results of a second study in Madinah indicated that, after applying taxes, soft drink consumption decreased by 19% among the participants [39]. The students’ practices in the current study showed another direction, as most of the participants stated that they still bought SSBs but less often and in smaller quantities after consuming SSBs. According to Minton, consumer behavior toward the impact of the SSB tax is unpredictable [40]. This may be because healthy options are usually more expensive than unhealthy options, in addition to their inability to stop consuming sugar due to its pleasurable and addictive properties [31]. This mechanism may be explained by sugar-releasing endogenous physiological opioids known as endorphins, which relieve pain and promote short-term euphoria [41,42]. Although the participants were aware that SSBs with high sugar levels were harmful to their health, it may have been difficult for them to stop consuming the SSBs. Hence, this does not negate the role and impact of SSB taxation in reducing consumption rates. Recent evidence suggests that taxes on SSBs have directly impacted consumption levels, particularly among low-income individuals and young adults, in addition to increasing the revenue across countries [39,43]. Similarly, a systematic review concluded that imposing a tax on SSBs effectively reduced consumption levels. Moreover, a review showed that taxes had a greater impact on low-income people [23]. In the United States, 36% of respondents supported a tax on SSBs [22]. The New York Survey also supported a previous study, with 52% of the respondents supporting a tax on SSBs, and the number of supporters increased to 72% when they learned that the tax revenue would be used to support obesity prevention and health promotion programs [44]. According to Myers et al., a tax on SSBs might be supported if individuals know that the revenue will be used to promote health [45]. Based on the SSB tax rates in Saudi Arabia (50% for soft drinks and 100% for energy drinks), it can be concluded that the SSB tax may affect the dietary habits of the community and that these changes may lead to good behavior. The WHO recommends the imposition of taxes on SSBs. Taxes may change individual behavior, leading to a lower consumption of SSBs, reducing health problems, and improving people’s lives in general. However, this does not negate the importance of awareness and education campaigns and implementing the SSB tax in reducing sugar consumption levels and increasing the awareness of good nutrition [39,46].

This study had several strengths and limitations. First, to the best of our knowledge, few studies have evaluated individuals’ KAPs concerning SSBs and their taxation in Saudi Arabia; thus, a strength of the current study lies in the fact that it examines this research gap. Second, the sample in this study was university students, who were among the highest consumers of SSBs, which confirmed the validity of selecting this target group for the current study. Finally, the questionnaire used in this study was previously tested and validated. Nevertheless, this study had several limitations. First, the cross-sectional study design only showed the correlations and not the causations. Second, online surveys for the data collection may have led to a selection bias; although, they are the most common method for assessing dietary patterns and have the advantages of ease and speed of communication, and low cost. Third, the questionnaire was self-reported by the participants, making it susceptible to bias. Finally, the target sample in this study was young adult students at King Abdulaziz University in Jeddah, Saudi Arabia; therefore, the findings may not be generalizable to the entire population of Saudi Arabia. However, these guides are believed to represent all university students in Jeddah because King Abdulaziz University is one of the largest and most prestigious universities in the country.

## 5. Conclusions

Our current study provided a foundation for understanding the KAPs concerning SSBs and the SSB taxation among students at King Abdulaziz University in Jeddah aged 18–25 years. Based on the study’s results, it can be concluded that 93% of the students have a high knowledge of SSBs, approximately 99% have positive attitudes towards SSBs, and 37% have good practices towards SSBs. The results also show that 73% of university students have a high knowledge of SSB taxation, 82% have positive attitudes towards SSB taxation, and approximately 32% perform good practices towards SSB taxation. The evidence indicates that there is a reasonable research gap between KAPs among university students and that it is necessary to implement interventions and strategies to transform the knowledge and attitudes into good practices and healthy lifestyle changes by reducing SSB consumption levels. Therefore, innovative strategies are urgently needed to reduce SSB consumption levels and promote good health among university students. Starting with the university environment for students, universities can limit the number of outlets selling SSBs; replace them with outlets selling healthy alternatives, such as fresh juices and snacks; and provide healthy alternatives at reasonable prices for students. Interventions should be at the community and individual levels, consider local culture, and promote healthy food choices among consumers. Thus, there is an urgent need for policies and strategic approaches to ensure public safety by reducing SSB consumption levels. These approaches include health education and nutrition awareness campaigns, addressing the issues of hidden sugars in foods and beverages, and educating the community on the names of different sugars commonly added to foods and beverages. Furthermore, taxes on SSBs should be augmented by supplementary legislations, such as promoting a cheaper and more affordable, healthy alternative to achieve the desired effects of the tax on reducing the consumption rates of SSBs. Finally, this study included only 380 university students. Therefore, we recommend that future studies to include a larger cohort of participants from various parts of Saudi Arabia for a more comprehensive understanding and broader generalization of the results. Importantly, further research should enhance our understanding of the KAPs related to SSBs and measure the long-term effects of the SSB tax. Moreover, subsequent investigations should examine the relationship between SSB consumption rates and BMI to obtain a greater understanding of the potential impacts of these beverages on weight status and overall health outcomes.

## Figures and Tables

**Table 1:** Socio-demographic characteristics and health data of the university students, stratified by gender.

Characterization	Total (n = 380) n (%)	Male (n = 184) n (%)	Female (n = 196) n (%)	X^2^	*p*-Value
Age					
18–19	135 (36)	63 (34)	72 (37)	6.93	0.074
20–21	134 (35)	75 (41)	59 (30)		
22–23	68 (18)	25 (14)	43 (22)		
24–25	43 (11)	21 (11)	22 (11)		
Age (Mean ± SD)	20.70 ± 1.90	20.65 ± 1.84	20.75 ± 1.96		0.617
Marital status					
Single	365 (96)	179 (97)	186 (94)	1.90	0.387
Married	14 (4)	5 (3)	9 (5)		
Divorced/widow	1 (0)	0 (0)	1 (1)		
Academic year					
First year (foundation year)	147 (39)	69 (37)	78 (40)	3.59	0.608
2nd year	89 (23)	40 (22)	49 (25)		
3rd year	57 (15)	34 (19)	23 (12)		
4th year	34 (9)	16 (9)	18 (9)		
5th year	33 (9)	15 (8)	18 (9)		
6th year	20 (5)	10 (5)	10 (5)		
College					
Health sciences faculties	23 (7)	9 (5)	14 (7)	22.12	<0.001
Sciences faculties	127 (33)	69 (38)	58 (30)		
Humanities and language faculties	104 (27)	37 (20)	67 (34)		
Environmental sciences faculties	22 (6)	19 (10)	3 (2)		
Foundation year	104 (27)	50 (27)	54 (27)		
Residence					
Living with family	362 (95)	172 (94)	190 (97)	2.51	0.113
Living alone	18 (5)	12 (6)	6 (3)		
Family income					
SAR < 5000	105 (28)	55 (30)	50 (26)	6.58	0.159
SAR 5000 to less than 10,000	82 (22)	37 (20)	45 (23)		
SAR 10,000 to less than 15,000	68 (18)	30 (16)	38 (19)		
SAR 15,000 to less than 20,000	65 (17)	26 (14)	39 (20)		
SAR > 20,000	60 (15)	36 (20)	24 (12)		
Smoking					
Yes	64 (17)	49 (27)	15 (8)	24.40	<0.001
No	316 (83)	135 (73)	181 (92)		
Activities or sports					
1–2 times a week	136 (36)	69 (37)	67 (34)	12.00	0.007
3–4 times a week	73 (19)	39 (21)	34 (17)		
>4 times a week	41 (11)	27 (15)	14 (7)		
None	130 (34)	49 (27)	81 (42)		
Sleep duration					
<5 h	48 (13)	25 (14)	23 (12)	0.34	0.842
5–8 h	271 (71)	129 (70)	142 (72)		
>8 h	61 (16)	30 (16)	31 (16)		
Sleep time					
At night	321 (85)	158 (86)	163 (83)	0.53	0.467
In the daytime	59 (15)	26 (14)	33 (17)		
Eating meals					
1 meal	47 (12)	18 (10)	29 (15)	14.22	0.001
2 meals	213 (56)	91 (49)	122 (62)		
3 meals	120 (32)	75 (41)	45 (23)		
BMI categories					
Underweight	57 (15)	20 (11)	37 (19)	13.43	0.004
Normal weight	204 (54)	91 (49)	113 (58)		
Overweight	77 (20)	46 (25)	31 (16)		
Obese	42 (11)	27 (15)	15 (7)		
BMI (Mean ± SD)	23.67 ± 6.00	24.61 ± 5.92	22.80 ± 5.96		0.003

The chi-squared test was used to examine differences between the two groups; a *p*-value of 0.05 was considered statistically significant.

**Table 2:** Socio-demographics associated with university students’ knowledge, attitudes, and practices concerning sugar-sweetened beverages.

Socio-Demographics	Knowledge				Attitudes				Practices			
	High Knowledgen = 352 n (%)	Low Knowledgen = 28 n (%)	X^2^	*p*-Value	Positive Attituden = 375 n (%)	Negative Attituden = 5 n (%)	X^2^	*p*-Value	Good Practicesn = 140 n (%)	Poor Practicesn = 240 n (%)	X^2^	*p*-Value
Gender												
Male	166 (47)	18 (64)	3.05	0.081	182 (48)	2 (40)	0.14	0.704	76 (54)	108 (45)	3.05	0.081
Female	186 (53)	10 (36)			193 (52)	3 (60)			64 (46)	132 (55)		
Age												
18–19	130 (37)	5 (18)	4.59	0.205	134 (36)	1 (20)	1.81	0.613	52 (37)	83 (35)	7.21	0.065
20–21	121 (34)	13 (46)			131 (35)	3 (60)			58 (41)	76 (32)		
22–23	61 (17)	7 (25)			67 (18)	1 (20)			19 (14)	49 (20)		
24–25	40 (12)	3 (11)			43 (11)	0 (0)			11 (8)	32 (13)		
Marital status												
Single	338 (96)	27 (96)	0.08	0.960	360 (96)	5 (100)	0.21	0.901	134 (96)	231 (96)	0.80	0.669
Married	13 (3)	1 (4)			14 (3)	0 (0)			6 (4)	8 (3)		
Divorced/widow	1 (1)	0 (0)			1 (1)	0 (0)			0 (0)	1 (1)		
Academic year												
First year (foundation year)	140 (40)	7 (25)	4.80	0.441	146 (39)	1 (20)	2.54	0.771	56 (40)	91 (38)	10.19	0.070
2nd year	83 (23)	6 (21)			87 (23)	2 (40)			32 (23)	57 (24)		
3rd year	50 (14)	7 (25)			56 (15)	1 (20)			27 (19)	30 (12)		
4th year	30 (9)	4 (14)			33 (9)	1 (20)			14 (10)	20 (8)		
5th year	30 (9)	3 (11)			33 (9)	0 (0)			5 (4)	28 (12)		
6th year	19 (5)	1 (4)			20 (5)	0 (0)			6 (4)	14 (6)		
College												
Health sciences faculties	22 (6)	1 (4)	3.84	0.428	23 (6)	0 (0)	11.02	0.026	7 (5)	16 (7)	2.89	0.576
Sciences faculties	114 (32)	13 (46)			126 (33)	1 (20)			51 (36)	76 (32)		
Humanities and language faculties	96 (27)	8 (29)			103 (28)	1 (20)			33 (24)	71 (29)		
Environmental sciences faculties	20 (6)	2 (7)			20 (5)	2 (40)			10 (7)	12 (5)		
Foundation year	100 (29)	4 (14)			103 (28)	1 (20)			39 (28)	65 (27)		
Residence												
Living with family	336 (95)	26 (93)	0.39	0.533	357 (95)	5 (100)	0.25	0.616	135 (96)	227 (95)	0.67	0.414
Living alone	16 (5)	2 (7)			18 (5)	0 (0)			5 (4)	13 (5)		
Family income												
SAR < 5000	97 (28)	8 (29)	0.32	0.988	105 (28)	0 (0)	4.41	0.354	48 (34)	57 (24)	13.64	0.009
SAR 5000 to less than 10,000	76 (22)	6 (21)			80 (21)	2 (40)			33 (24)	49 (20)		
SAR 10,000 to less than 15,000	64 (18)	4 (14)			66 (18)	2 (40)			24 (20)	40 (16)		
SAR 15,000 to less than 20,000	60 (17)	5 (18)			64 (17)	1 (20)			13 (9)	52 (22)		
SAR > 20,000	55 (15)	5 (18)			60 (16)	0 (0)			18 (13)	42 (18)		
Smoking												
Yes	59 (17)	5 (18)	0.02	0.881	61 (16)	3 (60)	6.74	0.009	29 (21)	35 (15)	2.37	0.123
No	293 (83)	23 (82)			314 (84)	2 (40)			111 (79)	205 (85)		
Activities or sports												
1–2 times a week	124 (35)	12 (43)	1.58	0.664	135 (36)	1 (20)	4.98	0.173	44 (32)	92 (38)	3.93	0.269
3–4 times a week	70 (20)	3 (11)			73 (19)	0 (0)			24 (17)	49 (20)		
>4 times a week	38 (11)	3 (11)			41 (11)	0 (0)			16 (11)	25 (11)		
None	120 (34)	10 (35)			126 (34)	4 (80)			56 (40)	74 (31)		
Sleep duration												
<5 h	44 (12)	4 (14)	0.66	0.719	46 (12)	2 (40)	3.91	0.142	20 (14)	28 (12)	1.30	0.522
5–8 h	250 (71)	21 (75)			286 (72)	3 (60)			95 (68)	176 (73)		
>8 h	58 (17)	3 (11)			61 (16)	0 (0)			25 (18)	36 (15)		
Sleep time												
At night	296 (84)	25 (89)	0.53	0.465	317 (85)	4 (80)	0.08	0.781	120 (86)	201 (84)	0.26	0.610
In the daytime	56 (16)	3 (11)			58 (15)	1 (20)			20 (14)	39 (16)		
Eating meals												
1 meal	44 (12)	3 (11)	1.78	0.410	46 (12)	1 (20)	2.36	0.308	13 (9)	34 (14)	2.53	0.282
2 meals	200 (57)	13 (46)			209 (56)	4 (80)			78 (56)	135 (56)		
3 meals	108 (31)	12 (43)			120 (32)	0 (0)			49 (35)	71 (30)		
BMI categories												
Underweight	53 (15)	4 (14)	1.59	0.661	55 (15)	2 (40)	3.74	0.291	20 (14)	37 (15)	1.05	0.789
Normal weight	191 (54)	13 (46)			201 (54)	3 (60)			72 (51)	132 (55)		
Overweight	71 (20)	6 (22)			77 (20)	0 (0)			32 (23)	45 (19)		
Obese	37 (11)	5 (18)			42 (11)	0 (0)			16 (12)	26 (11)		

The chi-squared test was used to examine differences between the two groups; a *p*-value of 0.05 was considered statistically significant.

**Table 3:** Socio-demographics associated with university students’ knowledge, attitudes, and practices concerning sugar-sweetened beverage taxation.

Socio-Demographics	Knowledge				Attitudes				Practices			
	High Knowledgen = 276 n (%)	Low Knowledgen = 104 n (%)	X^2^	*p*-Value	Positive Attituden = 311 n (%)	Negative Attituden = 69 n (%)	X^2^	*p*-Value	Good Practicesn = 122 n (%)	Poor Practicesn = 258 n (%)	X^2^	*p*-Value
Gender												
Male	122 (44)	62 (60)	7.19	0.007	149 (48)	35 (51)	0.18	0.672	49 (40)	135 (52)	4.91	0.27
Female	154 (56)	42 (40)			162 (52)	34 (49)			73 (60)	123 (48)		
Age												
18–19	102 (37)	33 (32)	2.93	0.402	108 (34)	27 (39)	3.08	0.379	40 (33)	95 (37)	4.38	0.223
20–21	96 (35)	38 (37)			114 (37)	20 (29)			45 (37)	89 (35)		
22–23	51 (18)	17 (16)			52 (17)	16 (23)			18 (15)	50 (19)		
24–25	27 (10)	16 (15)			37 (12)	6 (9)			19 (15)	24 (9)		
Marital status												
Single	264 (96)	101 (97)	3.88	0.144	297 (95)	68 (99)	1.42	0.492	118 (97)	247 (95)	0.56	0.755
Married	12 (4)	2 (2)			13 (4)	1 (1)			4 (3)	10 (4)		
Divorced/widow	0 (0)	1 (1)			1 (1)	0 (0)			0 (0)	1 (1)		
Academic year												
First year (foundation year)	110 (40)	37 (35)	2.16	0.829	117 (38)	30 (43)	6.04	0.302	43 (35)	104 (40)	2.41	0.790
2nd year	63 (23)	26 (25)			80 (26)	9 (13)			33 (27)	56 (22)		
3rd year	42 (15)	15 (14)			46 (15)	11 (16)			18 (15)	39 (15)		
4th year	25 (9)	9 (9)			26 (8)	8 (12)			9 (7)	25 (10)		
5th year	24 (9)	9 (9)			25 (8)	8 (12)			12 (10)	21 (8)		
6th year	12 (4)	8 (8)			17 (5)	3 (4)			7 (6)	13 (5)		
College												
Health sciences faculties	18 (7)	5 (5)	1.63	0.803	21 (7)	2 (3)	5.69	0.223	8 (7)	15 (6)	2.72	0.607
Sciences faculties	91 (33)	36 (34)			97 (31)	30 (43)			38 (31)	89 (34)		
Humanities and language faculties	75 (27)	29 (28)			88 (28)	16 (23)			39 (32)	65 (25)		
Environmental sciences faculties	14 (5)	8 (8)			20 (7)	2 (3)			5 (4)	17 (7)		
Foundation year	78 (28)	26 (25)			85 (27)	19 (28)			32 (26)	72 (28)		
Residence												
Living with family	261 (95)	101 (97)	1.09	0.297	294 (95)	68 (99)	2.01	0.155	113 (93)	249 (97)	2.78	0.096
Living alone	15 (5)	3 (3)			17 (5)	1 (1)			9 (7)	9 (3)		
Family income												
SAR < 5000	77 (28)	28 ± (27)	1.59	0.811	87 (28)	18 (26)	2.78	0.595	31 (26)	74 (29)	2.64	0.619
SAR 5000 to less than 10,000	57 (21)	25 (24)			64 (21)	18 (26)			27 (22)	55 (21)		
SAR 10,000 to less than 15,000	47 (17)	21 (20)			53 (17)	15 (22)			21 (17)	47 (18)		
SAR 15,000 to less than 20,000	49 (18)	16 (15)			56 (18)	9 (13)			26 (21)	39 (15)		
SAR > 20,000	46 (16)	14 (14)			51 (16)	9 (13)			17 (14)	43 (17)		
Smoking												
Yes	42 (15)	22 (21)	1.90	0.168	49 (16)	15 (22)	1.44	0.230	8 (7)	56 (22)	13.57	<0.001
No	234 (85)	82 (79)			262 (84)	54 (78)			114 (93)	202 (78)		
Activities or sports												
1–2 times a week	89 (32)	47 (45)	6.04	0.110	113 (36)	23 (33)	2.66	0.447	50 (41)	86 (33)	10.46	0.015
3–4 times a week	55 (20)	18 (17)			63 (20)	10 (15)			27 (22)	46 (18)		
>4 times a week	30 (11)	11 (11)			34 (11)	7 (10)			17 (14)	24 (9)		
None	102 (37)	28 (27)			101 (33)	29 (42)			28 (23)	102 (40)		
Sleep duration												
<5 h	38 (14)	10 (10)	1.93	0.380	39 (13)	9 (13)	0.01	0.994	17 (14)	31 (12)	0.43	0.806
5–8 h	197 (71)	74 (71)			222 (71)	49 (71)			87 (71)	184 (71)		
>8 h	41 (15)	20 (19)			50 (16)	11 (16)			18 (15)	43 (17)		
Sleep time												
At night	237 (86)	84 (81)	1.45	0.221	264 (85)	57 (83)	0.22	0.636	108 (89)	213 (83)	2.25	0.134
In the daytime	39 (14)	20 (19)			47 (15)	12 (17)			14 (11)	45 (17)		
Eating meals												
1 meal	39 (14)	8 (8)	4.27	0.118	36 (12)	11 (16)	1.73	0.421	11 (9)	36 (14)	1.92	0.382
2 meals	147 (53)	66 (63)			173 (55)	40 (58)			72 (59)	141 (55)		
3 meals	90 (33)	30 (29)			102 (33)	18 (26)			39 (32)	81 (31)		
BMI categories												
Underweight	43 (16)	14 (14)	3.13	0.372	46 (15)	11 (16)	0.75	0.862	19 (15)	38 (15)	5.62	0.132
Normal weight	153 (55)	51 (49)			170 (55)	34 (49)			72 (59)	132 (51)		
Overweight	50 (18)	27 (26)			62 (20)	15 (22)			24 (20)	53 (20)		
Obese	30 (11)	12 (11)			33 (10)	9 (13)			7 (6)	35 (14)		

The chi-squared test was used to examine differences between the two groups; a *p*-value of 0.05 was considered statistically significant.

**Table 4:** Association between knowledge, attitude, and practice scores concerning sugar-sweetened beverages and university students’ socio-demographic and health data stratified by gender.

Socio-Demographics	Male			Female		
	Knowledge	Attitudes	Practices	Knowledge	Attitudes	Practices
Age						
18–19	1.94 ± 0.25	2.00 ± 0.00	1.43 ± 0.5	1.99 ± 0.12	1.99 ± 0.12	1.35 ± 0.48
20–21	1.91 ± 0.29	1.99 ± 0.12	1.48 ± 0.5	1.90 ± 0.31	1.97 ± 0.18	1.37 ± 0.49
22–23	1.84 ± 0.37	1.96 ± 0.20	1.24 ± 0.44	1.93 ± 0.26	2.00 ± 0.00	1.30 ± 0.47
24–25	1.86 ± 0.36	2.00 ± 0.00	1.33 ± 0.48	2.00 ± 0.00	2.00 ± 0.00	1.18 ± 0.4
*p*-value	0.476	0.259	0.072	0.428	0.764	0.814
College						
Health sciences faculties	2.00 ± 0.00	2.00 ± 0.00	1.44 ± 0.53	1.93 ± 0.27	2.00 ± 0.00	1.21 ± 0.43
Sciences faculties	1.88 ± 0.32	2.00 ± 0.00	1.43 ± 0.5	1.91 ± 0.28	1.98 ± 0.13	1.36 ± 0.49
Humanities and language faculties	1.86 ± 0.35	2.00 ± 0.00	1.30 ± 0.46	1.96 ± 0.21	1.99 ± 0.12	1.33 ± 0.47
Environmental sciences faculties	1.89 ± 0.32	1.89 ± 0.32	1.47 ± 0.51	2.00 ± 0.00	2.00 ± 0.00	1.33 ± 0.58
Foundation year	1.94 ± 0.24	2.00 ± 0.00	1.44 ± 0.50	1.98 ± 0.14	1.98 ± 0.14	1.31 ± 0.47
*p*-value	0.742	0.004	0.547	0.442	0.970	0.349
Family income (SAR)						
SAR < 5000	1.93 ± 0.26	2.00 ± 0.00	1.51 ± 0.51	1.92 ± 0.27	2.00 ± 0.00	1.40 ± 0.5
SAR 5000 to less than 10,000	1.89 ± 0.32	1.97 ± 0.16	1.43 ± 0.50	1.96 ± 0.21	1.98 ± 0.15	1.38 ± 0.5
SAR 10,000 to less than 15,000	1.93 ± 0.25	1.97 ± 0.18	1.37 ± 0.5	1.95 ± 0.23	1.97 ± 0.16	1.45 ± 0.50
SAR 15,000 to less than 20,000	1.88 ± 0.33	2.00 ± 0.00	1.31 ± 0.47	1.95 ± 0.22	1.97 ± 0.16	1.13 ± 0.34
SAR > 20,000	1.86 ± 0.35	2.00 ± 0.00	1.36 ± 0.49	2.00 ± 0.00	2.00 ± 0.00	1.21 ± 0.42
*p*-value	0.679	0.358	0.333	0.263	0.695	0.017
Smoking						
Yes	1.90 ± 0.31	1.96 ± 0.20	1.47 ± 0.50	2.00 ± 0.00	1.93 ± 0.26	1.40 ± 0.51
No	1.90 ± 0.3	2.00 ± 0.00	1.39 ± 0.5	1.94 ± 0.23	1.99 ± 0.11	1.32 ± 0.47
*p*-value	0.811	0.022	0.145	0.365	0.113	0.416
Activities or sports						
1–2 times a week	1.87 ± 0.34	1.99 ± 0.12	1.41 ± 0.5	1.96 ± 0.21	2.00 ± 0.00	1.24 ± 0.43
3–4 times a week	1.92 ± 0.3	2.00 ± 0.00	1.28 ± 0.46	2.00 ± 0.00	2.00 ± 0.00	1.38 ± 0.5
>4 times a week	2.00 ± 0.00	2.00 ± 0.00	1.44 ± 0.51	1.79 ± 0.423	2.00 ± 0.00	1.29 ± 0.47
None	1.88 ± 0.33	1.98 ± 0.14	1.51 ± 0.51	1.95 ± 0.22	1.96 ± 0.2	1.38 ± 0.49
*p*-value	0.279	0.786	0.103	0.010	0.262	0.245
BMI categories						
Underweight	1.90 ± 0.31	1.95 ± 0.22	1.45 ± 0.51	1.95 ± 0.23	1.97 ± 0.16	1.30 ± 0.46
Normal weight	1.91 ± 0.29	1.99 ± 0.11	1.41 ± 0.5	1.96 ± 0.21	1.98 ± 0.13	1.31 ± 0.46
Overweight	1.89 ± 0.32	2.00 ± 0.00	1.41 ± 0.5	1.97 ± 0.18	2.00 ± 0.00	1.42 ± 0.50
Obese	1.89 ± 0.32	2.00 ± 0.00	1.41 ± 0.5	1.87 ± 0.35	2.00 ± 0.00	1.33 ± 0.49
*p*-value	0.989	0.351	0.855	0.311	0.826	0.642

Linear regression was used to examine the association between the socio-demographic characteristics, BMI, and knowledge, attitudes, and practices concerning sugar-sweetened beverage scores (*p* = 0.05).

**Table 5:** Association between knowledge, attitude, and practice scores for sugar-sweetened beverage taxation and university students’ socio-demographic and health data stratified by gender.

Socio-Demographics	Male			Female		
	Knowledge	Attitudes	Practices	Knowledge	Attitudes	Practices
Age						
18–19	1.68 ± 0.47	1.79 ± 0.41	1.24 ± 0.43	1.82 ± 0.39	1.81 ± 0.4	1.35 ± 0.48
20–21	1.69 ± 0.46	1.84 ± 0.37	1.29 ± 0.46	1.75 ± 0.44	1.86 ± 0.35	1.39 ± 0.49
22–23	1.68 ± 0.48	1.80 ± 0.41	1.16 ± 0.37	1.79 ± 0.41	1.74 ± 0.44	1.33 ± 0.47
24–25	1.48 ± 0.51	1.76 ± 0.44	1.38 ± 0.5	1.77 ± 0.43	1.95 ± 0.21	1.50 ± 0.51
*p*-value	0.357	0.551	0.219	0.494	0.094	0.284
College						
Health sciences faculties	1.67 ± 0.50	2.00 ± 0.00	1.22 ± 0.44	1.86 ± 0.36	1.86 ± 0.36	1.43 ± 0.51
Sciences faculties	1.67 ± 0.48	1.74 ± 0.44	1.28 ± 0.45	1.78 ± 0.42	1.79 ± 0.41	1.33 ± 0.47
Humanities and language faculties	1.59 ± 0.5	1.89 ± 0.32	1.35 ± 0.48	1.79 ± 0.41	1.82 ± 0.39	1.39 ± 0.49
Environmental sciences faculties	1.63 ± 0.5	1.89 ± 0.32	1.21 ± 0.42	1.67 ± 0.58	2.00 ± 0.00	1.33 ± 0.58
Foundation year	1.72 ± 0.45	1.78 ± 0.42	1.22 ± 0.42	1.78 ± 0.42	1.85 ± 0.36	1.39 ± 0.49
*p*-value	0.855	0.074	0.306	0.927	0.583	0.671
Family income (SAR)						
SAR < 5000	1.69 ± 0.47	1.84 ± 0.37	1.27 ± 0.45	1.78 ± 0.42	1.82 ± 0.39	1.32 ± 0.47
SAR 5000 to less than 10,000	1.59 ± 0.5	1.68 ± 0.48	1.35 ± 0.48	1.78 ± 0.42	1.87 ± 0.34	1.31 ± 0.47
SAR 10,000 to less than 15,000	1.53 ± 0.51	1.80 ± 0.41	1.20 ± 0.41	1.82 ± 0.39	1.76 ± 0.43	1.39 ± 0.5
SAR 15,000 to less than 20,000	1.73 ± 0.45	1.85 ± 0.37	1.19 ± 0.40	1.77 ± 0.43	1.87 ± 0.34	1.54 ± 0.51
SAR > 20,000	1.75 ± 0.44	1.89 ± 0.32	1.28 ± 0.45	1.79 ± 0.42	1.79 ± 0.42	1.29 ± 0.46
*p*-value	0.268	0.276	0.201	0.995	0.841	0.156
Smoking						
Yes	1.65 ± 0.48	1.80 ± 0.41	1.14 ± 0.35	1.67 ± 0.49	1.67 ± 0.49	1.07 ± 0.26
No	1.67 ± 0.47	1.81 ± 0.39	1.31 ± 0.47	1.80 ± 0.40	1.84 ± 0.37	1.40 ± 0.49
*p*-value	0.966	0.713	0.016	0.151	0.132	0.010
Activities or sports						
1–2 times a week	1.58 ± 0.5	1.81 ± 0.39	1.29 ± 0.46	1.73 ± 0.45	1.85 ± 0.36	1.45 ± 0.50
3–4 times a week	1.69 ± 0.47	1.87 ± 0.34	1.36 ± 0.49	1.82 ± 0.39	1.85 ± 0.36	1.38 ± 0.49
>4 times a week	1.67 ± 0.48	1.81 ± 0.4	1.33 ± 0.48	1.86 ± 0.36	1.86 ± 0.36	1.57 ± 0.51
None	1.76 ± 0.43	1.76 ± 0.43	1.12 ± 0.33	1.80 ± 0.40	1.79 ± 0.41	1.27 ± 0.45
*p*-value	0.289	0.513	0.050	0.432	0.811	0.057
BMI categories						
Underweight	1.55 ± 0.51	1.75 ± 0.44	1.20 ± 0.41	1.86 ± 0.35	1.84 ± 0.37	1.41 ± 0.5
Normal weight	1.75 ± 0.44	1.82 ± 0.38	1.29 ± 0.45	1.75 ± 0.43	1.84 ± 0.37	1.41 ± 0.49
Overweight	1.52 ± 0.51	1.83 ± 0.38	1.33 ± 0.47	1.84 ± 0.37	1.77 ± 0.43	1.29 ± 0.46
Obese	1.70 ± 0.47	1.78 ± 0.42	1.15 ± 0.36	1.73 ± 0.46	1.80 ± 0.41	1.20 ± 0.41
*p*-value	0.029	0.889	0.525	0.513	0.749	0.315

## Data Availability

The datasets generated and/or analyzed during this study are not publicly available owing to the use of data for further publications but are available from the corresponding authors on reasonable request.

## Supplementary Materials

The following supporting information can be downloaded at: https://www.mdpi.com/article/10.3390/nu15194151/s1, Table S1: university students’ knowledge regarding sugar-sweetened beverages; Table S2: university students’ attitudes regarding sugar-sweetened beverages; Table S3: university students’ practices regarding sugar-sweetened beverages; Table S4: university students’ knowledge regarding sugar-sweetened beverages taxation; Table S5: university students’ attitudes regarding sugar-sweetened beverages taxation; Table S6: university students’ practices regarding sugar-sweetened beverages taxation.
