# Supplementary material for: Understanding the Knowledge, Attitudes, and Practices Concerning Sugar-Sweetened Beverages and Beverage Taxation among Saudi University Students
Source: Nutrients. 2023 Sep 26;15(19):4151. doi: 10.3390/nu15194151 (PMC10574237; doi:10.3390/nu15194151)
Supplement: Supplementary file 1 [file nutrients-15-04151-s001.zip › nutrients-2574079-supplementary.pdf]

Section One: Demographic Information

This section aims to assess the sociodemographic characteristics of the study participants, and how they relate to knowledge and attitudes toward SSBs.

- Gender:

☐ Male
☐ Female

- Marital status:

☐ Single
☐ Married
☐ Divorced/widow

- Age

………………………………………………………

- Academic year:

☐ First year (preparatory year)
☐ Second year
☐ Third year
☐ Fourth year
☐ Fifth year
☐ Sixth year

- The college:

………………………………………………………

- University Specialization:

………………………………………………………

- Living:

☐ I live with family
☐ I live alone (University housing)

- The level of the monthly income of the family:

☐ Less than 5000 riyals
☐ From 5,000 to less than 10,000
☐ From 10,000 to less than 15,000
☐ From 15,000 to less than 20,000
☐ More than 20,000

- Are you a smoker? **(Includes smoke, shisha, electronic cigarette and equivalent)**

☐ Yes

□ No

- How many times per week do you do activities or sports?

□ 1-2 times a week
□ 3-4 times a week
□ More than 4 times a week
□ None

- How long do you usually sleep?

□ Less than 5 hours
□ 5-8 hours
□ More than 8 hours

- When do you usually sleep?

□ At night
□ In the daytime

- How many main meals you eat per day?

□ 1 meal
□ 2 meals
□ 3 meals

Section Two: Anthropometric Information

This section aims to examine the relationship between consumption of SSBs and participants' body mass index (BMI).

- Height …………………………………... m
- Weight …………………………………... kg

Section Three: Knowledge and attitudes toward sugar-sweetened beverages

The questions in this section aim to determine knowledge and attitudes towards SSBs among participating students at King Abdelaziz University. There are no right or wrong answers, and we value your answers **with credibility and transparency.**

- **Domain: knowledge**

No.	Items	Yes	No
1.	WHO recommends reducing the intake of simple sugar to less than 10% of total energy intake per day.		
2.	Intake of SSB is considered an appropriate strategy for increasing caloric intake in individuals with inadequate energy intake.		
3.	Dextrose is the scientific name of simple sugar.		
4.	15 g (1 tbsp) of sugar is equivalent to 15 g of carbohydrate in our diet.		
5.	Calories provided by 1 tbsp of condensed milk is equivalent to half tbsp of sugar.		
6.	A 250ml can of Energy Drink (Red Bull) is equivalent to 6.5 teaspoons of sugar.		
7.	Beverages with corn syrup stated in the list of ingredients classified as SSB.		
8.	SSB includes beverages with added honey.		
9.	Fresh fruit juices are classified as SSB.		
10	Plain chocolate drinks are classified as SSB.		
11.	Frequent consumption of SSBs increases the risk of obesity.		
12.	Frequent consumption of SSBs increases the risk of developing diabetes.		
13.	Frequent consumption of SSBs increases the risk of tooth decay.		
14.	Severe obese increases the risk of developing diabetes.		
15.	Severe obese increases the risk of heart disease.		
16.	Severe obese increases the risk of developing asthma.		
17.	Severe obese increases the risk of developing high blood pressure.		
18.	Severe obese increases the risk of developing cancer.		

- **Domain: attitudes**

No.	Items	Agree	Disagree
1.	It is important to read the list of the ingredients before choosing packed beverages in the market.		
2.	Consumers must know on how to read the label of foods or beverages especially with added sugar.		
3.	Choosing beverages without added sugar is much healthier compared to SSB.		

4.	Consumer should aware of other names of sugar that often added in food.		
5.	I need to have a good knowledge on reading the nutrition facts before choosing or buying foods or beverages in the market.		
6.	Consumers should know the health consequences of excessive consumption of SSBs such as obesity, diabetes, high blood pressure, heart disease and cancer.		

- **Domain: practice**

No.	Items	Yes	No
1.	I often identify the amount of sugar added in my drinks before consuming.		
2.	I often select flavoured milk compared to fresh milk.		
3.	I often compare the calorie contributed by the sugar added for each beverage before buying the item		
4.	I usually consumed SSBs only for breakfast.		
5.	I usually consume SSBs with fast food.		
6.	I usually consume 3 In 1 beverage as it is easy to prepare and convenient for my daily schedule.		
7.	I usually drink SSBs while (watching TV, cinema, playing electronic games or studying)		

Section Four: Knowledge and attitudes towards the tax on SSBs

In 2017, an added tax was introduced for SSBs. The questions in this section are intended to examine the knowledge and attitudes of the participants in this study toward the SSBs tax.

- What did you do when the SSBs tax was introduced (50% on soft drinks, 100% on energy drinks)?

☐ Replace it with non-taxable drinks (such as fresh fruit juices, water, or unflavored milk).
☐ Reduce consumption of SSBs (such as soft drinks and energy drinks).
☐ There is no change at all.

- Indicate your agreement that you are in favour of the Saudi Arabia SSBs tax.

☐ Strongly disagree
☐ Disagree
☐ Neutral
☐ Agree
☐ Strongly agree

- Please provide a reason for your response to the question above.

…………………………………………………………………………………………………
…………………………………………………………………………………………………
…………………………………………………………………………………………………

- From the options provided below, what would you most prefer the money generated from the sugar tax be used for? **(Select ONE option only)**

☐ Fundament-obesity interventions.
☐ Revenue for the government.
☐ Subsidizing healthy foods.
☐ Researching cures for diseases exacerbated by excess sugar consumption.

- Indicate your agreement/disagreement with the following statements:

No.	Statements	Strongly Disagree	Disagree	Neutral	Agree	Strongly Agree
1.	The sugar tax will help to reduce obesity in Saudi Arabia.					
2.	The Saudi Arabia sugar tax will help to reduce the consumption of SSBs amongst consumers.					
3.	The sugar tax is a money-generating scheme by the government					
4.	The Saudi Arabia sugar tax will negatively affect the economy (income).					
5.	The government has the right to influence purchasing decisions for SSBs in order to maintain health.					

- Indicate your response to the sugar tax by selecting **ONE** of the following practices that applies to you:

Following the introduction of the sugar tax on sugar-sweetened beverages, I will...

☐ Continue to buy SSB in the same quantity and with the same frequency that I did before the tax was introduced.
☐ Still buy SSB, but less often and in smaller quantities.
☐ Stop buying SSBs and rather buy some other beverage.
☐ This does not apply to me, as I do not buy SSB at all.

- If the sugar-sweetened beverages were no longer affordable to you, which alternatives would you consider buying? **(Tick ALL that apply)**

☐ This does not apply to me, as I do not buy SSB anyway.
☐ Milk and milk products.
☐ 100% fruit juice.

☐ Sugar-free drink options.
☐ Water.
☐ Other.

- If other was specified, please explain:

………
………

Table S1: University students' knowledge regarding sugar-sweetened beverages

Knowledge domain	Total (n = 380) n (%)
**WHO recommends reducing the intake of simple sugar to less than 10% of total energy intake per day.**	
Yes	285 ± 75
No	95 ± 25
**Intake of SSBs is considered an appropriate strategy for increasing caloric intake in individuals with inadequate energy intake.**	
Yes	145 ± 38
No	235 ± 62
**Dextrose is the scientific name of simple sugar.**	
Yes	160 ± 42
No	220 ± 58
**15 g (1 tbsp) of sugar is equivalent to 15 g of carbohydrate in our diet.**	
Yes	161 ± 42
No	219 ± 58
**Calories provided by 1 tbsp of condensed milk is equivalent to half tbsp of sugar.**	
Yes	168 ± 44
No	212 ± 56
**A 250ml can of Energy Drink (Red Bull) is equivalent to 6.5 teaspoons of sugar.**	
Yes	236 ± 62
No	144 ± 38
**Beverages with corn syrup stated in the list of ingredients classified as SSB.**	
Yes	235 ± 62
No	145 ± 38
**SSB includes beverages with added honey.**	
Yes	164 ± 43
No	216 ± 57
**Fresh fruit juices are classified as SSB.**	
Yes	147 ± 39
No	233 ± 61
**Plain chocolate drinks are classified as SSB.**	
Yes	330 ± 87
No	50 ± 13
**Frequent consumption of SSBs increases the risk of obesity.**	
Yes	353 ± 93
No	27 ± 7
**Frequent consumption of SSBs increases the risk of developing diabetes.**	
Yes	349 ± 92
No	31 ± 8
**Frequent consumption of SSBs increases the risk of tooth decay.**	
Yes	351 ± 92
No	29 ± 8

**Severe obese increases the risk of developing diabetes.**	
Yes	361 ± 95
No	19 ± 5
**Severe obese increases the risk of heart disease.**	
Yes	365 ± 96
No	15 ± 4
**Severe obese increases the risk of developing asthma.**	
Yes	240 ± 63
No	140 ± 37
**Severe obese increases the risk of developing high blood pressure.**	
Yes	350 ± 92
No	30 ± 8
**Severe obese increases the risk of developing cancer.**	
Yes	246 ± 65
No	134 ± 35

Table S2: University students' attitudes regarding sugar-sweetened beverages.

**Attitudes domain**	**Total (n = 380) n (%)**
**It is important to read the list of the ingredients before choosing packed beverages in the market.**	
Agree	355 ± 93
Disagree	25 ± 7
**Consumers must know on how to read the label of foods or beverages especially with added sugar.**	
Agree	361 ± 95
Disagree	19 ± 5
**Choosing beverages without added sugar is much healthier compared to SSB.**	
Agree	339 ± 89
Disagree	41 ± 11
**Consumer should aware of other names of sugar that often added in food.**	
Agree	334 ± 88
Disagree	46 ± 12
**I need to have a good knowledge on reading the nutrition facts before choosing or buying foods or beverages in the market.**	
Agree	336 ± 88
Disagree	44 ± 12
**Consumers should know the health consequences of excessive consumption of SSBs such as obesity, diabetes, high blood pressure, heart disease and cancer.**	
Agree	371 ± 98
Disagree	9 ± 2

Table S3: University students' practices regarding sugar-sweetened beverages.

Practice domain	Total (n = 380)<br>n (%)
**I often identify the amount of sugar added in my drinks before consuming.**	
Yes	208 ± 55
No	172 ± 45
**I often select flavoured milk compared to fresh milk.**	
Yes	123 ± 32
No	257 ± 68
**I often compare the calorie contributed by the sugar added for each beverage before buying the item.**	
Yes	162 ± 43
No	218 ± 57
**I usually consumed SSBs only for breakfast.**	
Yes	127 ± 33
No	253 ± 67
**I usually consume SSBs with fast food.**	
Yes	244 ± 64
No	136 ± 36
**I usually consume 3 In 1 beverage as it is easy to prepare and convenient for my daily schedule.**	
Yes	109 ± 29
No	271 ± 71
**I usually drink SSBs while (watching TV, cinema, playing electronic games or studying)**	
Yes	227 ± 60
No	153 ± 40

Table S4: University students' knowledge regarding sugar-sweetened beverages taxation.

Knowledge towards the tax on SSBs	Total (n = 380)<br>n (%)
**The sugar tax will help to reduce obesity in Saudi Arabia.**	
Strongly Disagree	48 ± 13
Disagree	55 ± 14
Neutral	77 ± 20
Agree	129 ± 34
Strongly Agree	71 ± 19
**The Saudi Arabia sugar tax will help to reduce the consumption of SSBs amongst consumers.**	
Strongly Disagree	30 ± 8
Disagree	51 ± 13
Neutral	64 ± 17
Agree	152 ± 40
Strongly Agree	83 ± 22

**The sugar tax is a money-generating scheme by the government.**	
Strongly Disagree	48 ± 13
Disagree	66 ± 17
Neutral	157 ± 41
Agree	70 ± 19
Strongly Agree	39 ± 10
**The Saudi Arabia sugar tax will negatively affect the economy (income).**	
Strongly Disagree	75 ± 20
Disagree	102 ± 27
Neutral	114 ± 30
Agree	65 ± 17
Strongly Agree	24 ± 6

Table S5: University students' attitudes regarding sugar-sweetened beverages taxation.

Attitudes towards the tax on SSBs	Total (n = 380) n (%)
**What did you do when the SSBs tax was introduced (50% on soft drinks, 100% on energy drinks)?**	
Replace it with non-taxable drinks (such as fresh fruit juices, water, or unflavored milk).	86 ± 23
Reduce consumption of SSBs (such as soft drinks and energy drinks).	129 ± 34
There is no change at all.	165 ± 43
**Indicate your agreement that you are in favour of the Saudi Arabia SSBs tax.**	
Strongly disagree	47 ± 12
Disagree	34 ± 9
Neutral	94 ± 25
Agree	96 ± 25
Strongly agree	109 ± 29
**From the options provided below, what would you most prefer the money generated from the sugar tax be used for?**	
Fundament-obesity interventions.	57 ± 15
Revenue for the government.	13 ± 4
Subsidizing healthy foods.	187 ± 49
Researching cures for diseases exacerbated by excess sugar consumption.	123 ± 32
**The government has the right to influence purchasing decisions for SSBs in order to maintain health.**	
Strongly Disagree	42 ± 11
Disagree	42 ± 11
Neutral	121 ± 32
Agree	97 ± 26
Strongly Agree	78 ± 20

Table S6: University students' practices regarding sugar-sweetened beverages taxation.

Practices towards the tax on SSBs	Total (n = 380) n (%)
**Following the introduction of the sugar tax on sugar-sweetened beverages, I will...**	
Continue to buy SSB in the same quantity and with the same frequency that I did before the tax was introduced	56 ± 15
Still buy SSB, but less often and in smaller quantities.	202 ± 53
Stop buying SSBs and rather buy some other beverage.	30 ± 8
This does not apply to me, as I do not buy SSB at all.	92 ± 24
**If the sugar-sweetened beverages were no longer affordable to you, which alternatives would you consider buying?**	
This does not apply to me, as I do not buy SSB anyway	75 ± 20
Milk and milk products	158 ± 41
100% fruit juice	32 ± 8
Sugar-free drink options	45 ± 12
Water	64 ± 17
Other.	6 ± 2
